# β2-Glycoprotein I-Reactive T Cells in Autoimmune Disease

**DOI:** 10.3389/fimmu.2018.02836

**Published:** 2018-12-10

**Authors:** Joyce Rauch, David Salem, Rebecca Subang, Masataka Kuwana, Jerrold S. Levine

**Affiliations:** ^1^Division of Rheumatology, Department of Medicine, Research Institute of the McGill University Health Centre, Montreal, QC, Canada; ^2^Department of Allergy and Rheumatology, Graduate School of Medicine, Nippon Medical School, Tokyo, Japan; ^3^Section of Nephrology, Department of Medicine, University of Illinois at Chicago and Section of Nephrology, Department of Medicine, Jesse Brown Veterans Affairs Medical Center, Chicago, IL, United States

**Keywords:** β2-glycoprotein I, T cells, systemic lupus erythematosus, anti-phospholipid syndrome, autoantibodies, MHC class II haplotypes

## Abstract

Anti-phospholipid syndrome (APS) and systemic lupus erythematosus (SLE) are autoimmune diseases characterized by autoantibody production and autoantibody-related pathology. Anti-phospholipid antibodies (aPL) are found in all patients with APS and in 20–30% of individuals with SLE. aPL recognize a number of autoantigens, but the primary target in both APS and SLE is β2-glycoprotein I (β2GPI). The production of IgG aPL in APS and SLE, as well as the association of aPL with certain MHC class II molecules, has led to investigation of the role of β2GPI-reactive T helper (Th). β2GPI-reactive CD4 Th cells have been associated with the presence of aPL and/or APS in both primary APS and secondary APS associated with SLE, as well as in SLE patients and healthy controls lacking aPL. CD4 T cells reactive with β2GPI have also been associated with atherosclerosis and found within atherosclerotic plaques. In most cases, the epitopes targeted by autoreactive β2GPI-reactive CD4 T cells in APS and SLE appear to arise as a consequence of antigenic processing of β2GPI that is structurally different from the soluble native form. This may arise from molecular interactions (e.g., with phospholipids), post-translational modification (e.g., oxidation or glycation), genetic alteration (e.g., β2GPI variants), or molecular mimicry (e.g., microbiota). A number of T cell epitopes have been characterized, particularly in Domain V, the lipid-binding domain of β2GPI. Possible sources of negatively charged lipid that bind β2GPI include oxidized LDL, activated platelets, microbiota (e.g., gut commensals), and dying (e.g., apoptotic) cells. Apoptotic cells not only bind β2GPI, but also express multiple other cellular autoantigens targeted in both APS and SLE. Dying cells that have bound β2GPI thus provide a rich source of autoantigens that can be recognized by B cells across a wide range of autoantigen specificities. β2GPI-reactive T cells could potentially provide T cell help to autoantigen-specific B cells that have taken up and processed apoptotic (or other dying) cells, and subsequently present β2GPI on their surface in the context of major histocompatibility complex (MHC) class II molecules. Here, we review the literature on β2GPI-reactive T cells, and highlight findings supporting the hypothesis that these T cells drive autoantibody production in both APS and SLE.

## Introduction

Systemic lupus erythematosus (SLE) is a heterogeneous autoimmune disease in which individuals develop multiple different autoantibodies, as well as a diversity of organ-related pathologies (1–3). In contrast, anti-phospholipid syndrome (APS) is a more homogeneous syndrome, with a limited number of autoantibodies and pathological outcomes (4, 5). Anti-phospholipid antibodies (aPL) are a key feature in both APS and SLE (4, 5). They are found in all patients with APS and in 20–30% of patients with SLE (6). Among SLE patients, autoantibodies including aPL can be detected up to 10 years before diagnosis (7). Remarkably, SLE autoantibodies targeting a multitude of cellular antigens emerge in a sequential order, with aPL being among the very first (7, 8). While the connection between aPL and autoimmune disease remains strongest for SLE and APS, aPL have also been linked to other autoimmune diseases, such as rheumatoid arthritis (RA) (9). In an inception cohort of patients with connective tissue diseases, the prevalence of aPL was similar for SLE and RA patients at ~15.7% (9). As in SLE and APS, autoantibodies precede the diagnosis of RA by several years (10). A common feature among APS, SLE, and RA that may help to understand the transition from serologic to pathologic autoimmunity is altered IgG glycosylation (11–14). For example, in RA, IgG glycosylation was similar in patients and controls a decade prior to the diagnosis of RA, but altered substantially ~3.5 years before disease onset (12). Taken together, these findings suggest a common mechanism for autoantibody generation and progression to organ pathology in autoimmune disease, and one in which aPL may be key, particularly APS and SLE. Although APS and SLE differ in their clinical manifestations, there is significant overlap in individuals affected by both diseases (6). Indeed, APS has been shown to develop in 50–70% of patients with aPL-positive SLE patients after 20 years of follow-up (6).

Both APS and SLE are characterized by the production of high levels of IgG class-switched autoantibodies, consistent with a T helper (Th) cell response. In APS, the autoantibodies primarily recognize phospholipid-binding proteins, such as β2-glycoprotein I (β2GPI) and prothrombin. In SLE, the range of autoantibodies is much broader, and includes aPL as well as autoantibodies targeting non-protein antigens, such as double-stranded DNA (dsDNA). In both APS and SLE, β2GPI is the primary target of the aPL. One of the major gaps in our understanding of SLE is how a T cell response can develop to a non-protein antigen. It has been noted that many of the non-protein autoantigens (e.g., DNA, RNA, phospholipid) targeted in SLE form complexes *in vivo* with protein antigens (1). This has led to speculation that a T cell response to the protein portion of the complex may provide T cell help to the complex's non-protein entity via intermolecular epitope spread. For example, a “hapten-carrier” model has been proposed to explain the production of anti-DNA autoantibodies in SLE (15). In this model, DNA is the “hapten” (i.e., non-immunogenic molecule) and elicits an immune response only when bound to a DNA-binding “carrier protein” (i.e., immunogenic molecule), such as histones, which can activate functional Th cells (15).

Our group has proposed a similar “hapten-carrier” model to address the breadth of the autoantibody response in SLE, in which an apoptotic or other dying cell—in particular, its non-protein determinants (e.g., phospholipid or DNA)—serve as “haptens,” while β2GPI serves as the “carrier protein” and promotes the activation of β2GPI-reactive T cells (16). In this regard, the phospholipid-binding property of β2GPI is critical, as it enables β2GPI to bind to the negatively charged surface of apoptotic cells, as well as other negatively charged particles and molecules (17). The ability of β2GPI to interact with dying cells is of particular relevance to this review (18–20). Apoptotic cells have long been proposed as a source of autoantigens in SLE (16, 21–23), and the physical interaction of β2GPI with these cells provides a “carrier protein”-like connection to a large pool of cellular autoantigens. β2GPI-reactive T cells therefore have the potential to promote autoantibody production to a multitude of self-antigens expressed by dying cells (24). Here, we review the literature and present findings supporting the hypothesis that β2GPI-reactive T cell responses stimulate autoantibody production in both APS and SLE.

## β2GPI-Reactive T Cells in APS and SLE

### Overview

Evidence of a role for Th cells in APS comes from the association of aPL with certain MHC class II genes (25), as well as from autoantibody class-switch to IgG. Similarly, Th cells are implicated (26) in the pathophysiology of SLE by virtue of both MHC class II associations (27) and IgG autoantibody production (2), as well as aberrant signaling defects reported in SLE T cells (28). Multiple HLA alleles, including HLA-DR2 and HLA-DR3, are associated with SLE, but the strength of this association and the specific allele(s) identified depend on the ethnic group and clinical presentation studied (29). The lack of consistent MHC class II associations in SLE, and the multitude of autoantigens targeted, make identification of critical Th cell epitopes in this disease a major challenge. Additional evidence of the importance of Th cells in these diseases derives from murine models. Anti-CD4 antibodies prevented disease in a model of SLE with APS (30), and bone marrow cells transferred experimental APS to naive mice only when T cells were present (31).

Interest in β2GPI-reactive Th cells developed in the late 1990's to early 2000's (32–35), about 10 years after the discovery that β2GPI, and not phospholipid, was the antigen recognized by anti-cardiolipin antibodies (anti-CL) (36, 37). Most published studies on human β2GPI-reactive T cells include both primary and secondary APS patients, as well as SLE patients without APS. Hence, it is difficult to discuss findings for β2GPI-reactive T cells in APS patients separately from SLE patients without APS. For this reason, we will discuss β2GPI-reactive T cells in APS and SLE concurrently. In this way, findings (often within the same study) for the different disease groups and subsets can be compared.

### Association of β2GPI-Reactive T Cells With Autoantibodies and Disease

In early studies of β2GPI-reactive T cells, patients were usually classified according to aPL reactivity or to the presence vs. absence of APS. Many of these studies evaluated T cell reactivity using peripheral blood mononuclear cells (PBMCs) from patients and healthy individuals, while others used patient-derived T cell lines or clones. Visvanathan et al. (33) studied the response of PBMCs to native plasma-derived β2GPI using a serum-free system in 24 aPL-positive (anti-CL- or lupus anticoagulant [LA]-positive) individuals, 7 aPL-negative individuals with various autoimmune diseases (including SLE), and 15 healthy controls. Of the 24 aPL-positive individuals, 18 had APS (5 SLE, 13 primary APS) and the remaining 6 autoimmune patients lacked clinical manifestations of APS (only one with SLE). PBMC responses to β2GPI were observed only in the aPL-positive group, and specifically in patients with APS (8 out of 18, 4 with SLE, and 4 with primary APS). Statistically, PBMC responses were associated with a history of APS, but not with IgG anti-β2GPI levels, and were characterized by a selective expansion of CD4 T cells producing IFN-γ, but not IL-4 (Th1-like response) (33).

Hattori et al. (34) also studied the PBMC responses in APS patients (5 SLE, 7 primary APS) and in SLE patients without APS (*n* = 13), as well as in healthy controls (*n* = 12). In contrast to Visvanathan et al. (33), they used dithiothreitol-reduced, not native, β2GPI as the stimulating antigen, and β2GPI-depleted serum in the culture medium. Moreover, patients were analyzed according to anti-β2GPI IgG antibody reactivity. PBMC responses to β2GPI were found in all anti-β2GPI-positive patients (6 primary APS, 4 SLE with APS, 2 SLE without APS), but also in anti-β2GPI-negative individuals (4 SLE without APS, 6 healthy controls). Most (91%) individuals with PBMC responses to β2GPI (“responders”) expressed HLA-DR53-associated alleles (DRB1^*^04, ^*^07, or ^*^09), as compared to 47% of “non-responders.” The domain specificity of the CD4 T cell proliferative response to recombinant β2GPI was assessed in six patients positive for anti-β2GPI antibodies (3 primary APS, 2 SLE with APS, 1 SLE without APS), and all recognized an epitope within Domains IV and/or V. Patients with the DRB1^*^09:01; DQB1^*^03:03 haplotype also recognized an epitope within Domains III/IV, while T cells from patients not expressing this haplotype recognized only Domains IV/V. Finally, T cells from one primary APS patient recognized Domains I/II as well as Domain IV/V.

To further analyze the epitope specificity and functional capacity of the T cells in these patients, Arai et al. (38) generated CD4 T cell clones from three patients with APS (2 primary APS, 1 SLE with APS). The majority (6 out of 7) of the β2GPI-specific T cell clones recognized a peptide encompassing amino acid residues 276–290 (KVSFFCKNKEKKCSY) in Domain V of β2GPI in the context of the DRB4^*^01:03 allele. Interestingly, this peptide spans the major phospholipid-binding site of β2GPI. All of the β2GPI-reactive T cell clones produced IFN-γ and had a Th1- or Th0-like cytokine expression profile. While the majority (10 of 12) of the β2GPI-specific T cell clones stimulated autologous peripheral blood B cells to produce anti-β2GPI antibodies *in vitro*, IFN-γ was not involved in B cell activation by these clones. Instead, stimulation was dependent on T cell production of IL-6 and CD40-CD40L interaction. The authors suggest that IL-6 and CD40L could be targeted therapeutically in APS patients resistant to anticoagulation.

T cell receptor (TCR) β chain usage was also analyzed in individuals demonstrating PBMC responses to β2GPI (5 APS patients and 3 healthy controls) (39). Vβ7 and Vβ8 were the most commonly detected TCRβ chains, and T cells expressing these two chains exhibited limited complementarity-determining region 3 (CDR3) sequence diversity. The Vβ7 chain was used by β2GPI-reactive T cells in PBMCs from 5 of 5 patients with APS, and 2 of 3 healthy controls. These findings from a limited group of individuals suggest a preferential usage of TCRβ chains by β2GPI-reactive T cells, whether in APS or healthy individuals.

Ito et al. (35) also investigated T cell responses to β2GPI in PBMCs from 18 patients (1 primary APS, 4 SLE with APS, 10 SLE without APS, and 3 SLE-like without APS) and 10 healthy controls. Instead of full-length β2GPI or its intact domains, they used a peptide library encompassing the full β2GPI sequence to screen PBMCs. Four patients and 2 controls had positive responses, and their T cells were used to generate 7 CD4 T cell lines that did not respond to native plasma-derived β2GPI. A limited number of epitopes were observed. Three of the 4 patient-derived T cell lines recognized peptide 244–264 in Domain V; this same peptide was also recognized by a cell line derived from a healthy control. The other peptides that were recognized were 64–83, 154–174, and 226–246. No association was observed between peptide recognition and a particular HLA class II molecule. Interestingly, however, cytokine production differed significantly between patient- and control-derived T cell lines. Although both produced IL-4 and IFN-γ, patient-derived T cell lines had significantly lower IFN-γ/IL-4 ratios than control lines, primarily due to lower IFN-γ responses in the patient-derived lines. Of note, none of the T cell lines reacted with native β2GPI. Together, these findings indicate that β2GPI-reactive CD4 T cell lines from this group of APS and SLE patients predominantly recognize the 244–264 epitope within Domain V of β2GPI. They do so in the context of various HLA class II molecules, and exhibit Th0- or Th2-like responses. In contrast, T cell lines from healthy controls display a Th0- or Th1-like phenotype.

Important methodological differences exist among the studies summarized to this point. The discovery of β2GPI-reactive T cells among anti-β2GPI- and APS-negative individuals (both SLE patients and healthy controls) by Hattori et al. (34) and Ito et al. (35), but not by Visvanathan et al. (33), may be attributed to such differences. To evaluate PBMC reactivity, Hattori et al. (34) used chemically reduced β2GPI, whereas Ito et al. (35) used a peptide library, and Visvanathan et al. (33) used native β2GPI. The decision of Hattori et al. (34) to use chemically reduced β2GPI was based on their observation that patient PBMCs did not respond to tissue culture medium with 10% human serum containing native β2GPI. While secondary cultures of PBMCs responsive to reduced β2GPI also recognized full-length recombinant β2GPI, they still lacked reactivity to native β2GPI. Similarly, Ito et al. (35) chose to evaluate synthetic β2GPI peptides because their T cell lines did not respond to native β2GPI isolated from human plasma. In contrast, Visvanathan et al. (33), using a serum-free system, showed that PBMCs from APS patients responded both to purified plasma-derived β2GPI and to native β2GPI in whole plasma. A second major difference between the studies was patient selection. Hattori et al. (34) and Ito et al. (35) selected patients based on a clinical diagnosis of SLE or APS (primary or secondary), while Visvanathan et al. (33) selected patients based on laboratory criteria for aPL (defined in that study as IgG or IgM anti-CL, or LA). Third, the geographical and, likely, the ethnic origin of individuals in the studies by Hattori et al. and Ito et al. differed from that in the study by Visvanathan et al.: Japan (34, 35) and Australia (33), respectively. Finally, Visvanathan et al. (33) evaluated neither the HLA association of the PBMC response nor its epitope specificity. The studies from Hattori et al. (34) and Ito et al. (35), while having many similarities, also exhibit subtle differences. Epitope specificity, although primarily within Domain V for both groups, differed in its precise mapping (35, 38). The pattern of cytokine production also differed; it was Th1-like for healthy controls in both studies (34), but Th1-like vs. Th2-like for patients in studies by Ito et al. (35) and Hattori et al. (34), respectively. The β2GPI T cell epitopes identified in the studies by Ito et al. (35) and Arai et al. (38) are shown in Table 1, and compared with epitopes identified in later studies (as discussed below).

**Table 1:** β2GPI CD4^+^ T cell epitopes identified in APS and SLE.

**Peptide sequence**	**Domain**	**Disease**	**Source (MHC class II)**	**Publication**
^1^MISPVLILFSSFLCHVAIAG^20^	I	APS	Human PBMCs (DRB3*02:02)^†^	de Moerloose et al. (42)
^26^PDDLPFSTVVPLKTF^40^	I	Induced SLE	129S1 (I-A^b^)	Salem et al. (40)
^31^FSTVVPLKTFYEPGE^45^	I	Induced SLE	BALB/c (I-A^d^/I-E^d^)	Salem et al. (40)
^111^NTGFYLNGADSAKCT^125^	II	PAPS Induced SLE	Human PBMCs (DRB1*04:03) C57BL/6 (I-A^b^), 129S1(I-A^b^)	Salem et al. (40)
^154^ECLPQHAMFGNDTITCTTHGN^174^	III	SAPS	Human PBMCs 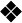	Ito et al. (35)
^159^SAGNNSLYRDTAVFECLP^176^	III	Induced SLE Spontaneous SLE	C57BL/6 (I-A^b^), C3H/HeN (I-A^k^/I-E^k^) MRL/lpr (I-A^k^/I-E^k^)	Salem et al. (41)
^165^LYRDTAVFECLPQHAMFG^182^	III	Induced SLE Spontaneous SLE	C57BL/6 (I-A^b^), C3H/HeN (I-A^k^/I-E^k^) MRL/lpr (I-A^k^/I-E^k^)	Salem et al. (41)
^208^PSRPDNGFVNYPAKPTLY^225^	IV	Induced SLE Spontaneous SLE	C3H/HeN (I-A^k^/I-E^k^) MRL/lpr (I-A^k^/I-E^k^)	Salem et al. (41)
^256^AMPSCKASCKVPVKKATV^273^	IV/V	Induced SLE	C3H/HeN (I-A^k^/I-E^k^)	Salem et al. (41)
^244^SCKLPVKKATVVYQGERVKIQ^264^	V	SAPS, SLE	Human PBMCs (DRB1*04:03, DRB4*01:03)^†^	Ito et al. (35)
^247^VPVKKATVVYQGERV^261^	V	PAPS	Human PBMCs (DRB1*04:03, DRB4*01:03)	Arai et al. (38)
^276^KVSFFCKNKEKKCSY^290^	V	PAPS, SAPS	Human PBMCs (DRB4*01:03)	Arai et al. (38)

APS, anti-phospholipid syndrome; SAPS, secondary APS; SLE, systemic lupus erythematosus. The numbering of amino acids in the studies by Salem et al. (40, 41) and de Moerloose et al. (42) includes the 19-amino acid leader sequence. In studies by Salem et al. (40, 20), murine T cells were derived from spleen.

† *Prominent HLA restrictions are noted here, but additional restrictions were found; 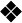, HLA restriction was not defined*.

Davies et al. (43) directly evaluated whether a PBMC response to native β2GPI was associated with the presence of anti-β2GPI and/or specific MHC class II genotypes in a cohort of Caucasian SLE patients in England. They found a proliferative PBMC response to β2GPI in 15/51 (29%) SLE patients, compared to 7% of controls. Proliferative responses to β2GPI were observed in SLE patients in the presence or absence of anti-β2GPI antibodies; however, some of the anti-β2GPI-negative patients had anti-CL. Patients with anti-β2GPI and/or anti-CL had a significantly higher proliferative response, compared to healthy controls. Despite the fact that certain HLA genotypes were associated with the presence of anti-β2GPI, no association was found between proliferative PBMC responses to β2GPI and any HLA genotypes.

A more recent study comparing PBMC responses to native β2GPI in unselected SLE and primary APS patients found a similar frequency in both groups (32% [12/37] in SLE vs. 25% [3/12] in primary APS) and no response in 23 control subjects (44). Recruitment of both SLE and primary APS patients was consecutive, and 38% of SLE patients had secondary APS. PBMCs proliferating to native β2GPI produced IFN-γ, but not IL-4. Proliferation was statistically associated with all of the following: IgM anti-β2GPI and anti-CL levels, a history of arterial thrombosis, and increased intimal-medial thickness. Interestingly, PBMC proliferation to β2GPI was also associated with a history of anti-nuclear antibodies and anti-dsDNA serum positivity, indicating that β2GPI-reactive T cells can be associated with SLE autoantibodies other than aPL.

Few other studies have addressed the relevance of immune reactivity to β2GPI for autoantibodies other than aPL in human SLE. Arbuckle et al. (7) showed that anti-CL (i.e., β2GPI-reactive antibodies) are among the earliest autoantibodies to appear in individuals who develop SLE, and can appear up to 7.6 years before diagnosis. The same group (8) further showed that, among SLE patients, individuals who developed anti-CL prior to diagnosis of SLE had a more severe and complex clinical outcome than individuals lacking anti-CL. Patients who were anti-CL positive prior to diagnosis presented with a greater number of classification criteria for SLE, compared to other SLE patients (6.1 vs. 4.9 criteria, *P* < 0.001). Disease onset occurred almost 4 years earlier in anti-CL-positive SLE patients, with earlier onset of such clinical manifestations as malar rash, photosensitivity, serositis, neurologic symptoms, and nephropathy. In addition, SLE-specific autoantibodies appeared earlier in aCL-positive individuals. Anti-dsDNA and anti-Sm antibodies appeared ~1 and 2 years earlier, respectively, and anti-dsDNA antibodies occurred more frequently (79% vs. 55% in anti-CL-negative individuals). Although not evaluated in this cohort of SLE patients, it seems likely that these individuals had β2GPI-reactive T cells, given the association between anti-CL and a T cell response to β2GPI observed in other studies (33, 44).

Taken together, these findings suggest that a cellular immune response to β2GPI exists in patients having both APS and SLE across a wide spectrum of MHC class II genotypes, and is associated with autoantibodies other than those reactive with β2GPI. While the frequency and clinical/serological associations vary among studies, these differences may be attributed to a number of factors, including patient selection and the nature of the antigen (native, reduced, or recombinant β2GPI; or peptide library) used to evaluate T cell reactivity. The presence of β2GPI-reactive T cells in healthy controls also varies among studies, but seems more frequent in studies not using native β2GPI.

### The Antigenic Stimulus for β2GPI-Reactive T Cells

#### Structural Alteration of Self-Antigen

Notably, many β2GPI-reactive T cells derived from PBMCs do not respond to native β2GPI, but respond well to bacterially expressed recombinant β2GPI fragments and to chemically reduced β2GPI. These findings suggest that the generation of β2GPI T cell epitopes requires unfolding or structural modification of β2GPI. Kuwana et al. (45) demonstrated that anionic phospholipid may be involved in the generation of T cell epitopes (often referred to as “cryptic epitopes”) not generated through processing of native β2GPI. They showed that dendritic cells or macrophages pulsed with vesicles containing anionic phospholipid and β2GPI, but not β2GPI or phospholipid alone, induced a response in human T cell lines specific for the Domain V epitope (276–290) in an HLA-DRB4^*^01:03-restricted manner. A later study showed that the same epitope can be generated *in vivo* by monocytes through FcγRI-mediated uptake of negatively charged particles (e.g., phosphatidylserine-containing vesicles) that have bound β2GPI in the presence of IgG anti-β2GPI antibodies (46). β2GPI bound to oxidized LDL or activated platelets also induced β2GPI-specific T cell responses (46). These data suggest that disease-relevant T cell epitopes in β2GPI may arise as a consequence of antigen processing of anionic phospholipid-bound β2GPI.

Buttari and coworkers (47, 48) also demonstrated that modification of β2GPI enhanced T cell activation, but they used alloreactive, rather than β2GPI-specific, T cells. They found that oxidized (47) and glucose-modified (48) β2GPI not only activated immature monocyte-derived dendritic cells from healthy human donors, but also increased allostimulatory ability in a mixed lymphocyte reaction. Dendritic cells activated by these modified forms of β2GPI also primed naïve allogeneic T cells, and induced Th polarization (primarily Th1-like for oxidized β2GPI, and Th2-like for glucose-modified β2GPI) (47, 48). These investigators suggest that oxidized β2GPI leads to dendritic cell maturation via interaction with a toll-like receptor (TLR), while glucose-modified β2GPI utilizes a receptor for advanced glycation end products.

Conformational changes in β2GPI resulting from genetic variants of the protein can also induce stronger T cell responses. For example, the V^247^ polymorphism located on exon 7, which leads to a substitution of leucine (L) for valine (V) at amino acid position 247 in Domain V of β2GPI, is associated with high titers of anti-β2GPI and arterial thrombosis in Mexican patients with primary APS (49). Genotypes for β2GPI can be VL, VV, or LL. Núñez-Álvarez et al. (50) assessed the proliferative response of PBMCs to the VL, VV, and LL isoforms at position 247 of β2GPI in 10 primary APS patients and 10 healthy individuals. PBMCs from primary APS patients had a stronger proliferative response than healthy controls to the VV and VL isoforms of β2GPI, but not to the LL isoform. The strongest response was to the VL form of β2GPI. Proliferation was stronger to chemically reduced vs. native isoforms, particularly for VV. The proliferative response of healthy control PBMCs was much lower than that of primary APS patients, and it did not appear to differentiate between isoforms or reduced/native conditions. Núñez-Álvarez et al. (50) further showed using differential scanning calorimetry that the structures of the V^247^ and the L^247^ isoforms of β2GPI differ, indicating that a single amino acid change at position 247 results in a major conformational change in β2GPI.

Together, these findings suggest that structural changes in β2GPI resulting from molecular interactions (e.g., with phospholipids), post-translational modification (e.g., oxidation or glycation), or genetic alteration (e.g., β2GPI variants) can enhance the presentation of disease-relevant epitopes. It is noteworthy that in a large retrospective multicenter analysis, patients with APS had higher levels of both native β2GPI and oxidized β2GPI than control groups including healthy individuals, autoimmune disease controls (with or without aPL, but lacking APS), and patients with thrombosis but no aPL (51, 52). Krilis and coworkers have proposed that post-translationally modified β2GPI can break immune tolerance, either because the modified form of β2GPI is not represented in the thymus or because intracellular processing of the oxidized form of β2GPI is different from that of the circulating (reduced) protein (51, 52). The latter hypothesis is consistent with the current evidence that some β2GPI-reactive T cells respond to modified, but not, native β2GPI.

#### Molecular Mimicry

The microbiome may potentially be a source of self-antigens that either trigger or perpetuate an autoreactive T cell response in APS (53) and SLE (54–56). Ruff et al. (53) have proposed that commensal bacteria act as a reservoir of cross-reactive antigen in APS and SLE through a mechanism called “molecular mimicry.” Molecular mimicry occurs when B and/or T cells responding to microbial pathogens also recognize (cross-react with) self-antigen. Ruff and coworkers (53) have identified peptides that are potentially cross-reactive with dominant T cell epitopes in APS in *Roseburia intestinalis*, a gram-positive anaerobic commensal particularly abundant in the human gut and stimulatory to lymphocytes from APS patients [unpublished observations in Ruff et al. (53)]. In SLE patients, skin and mucosal commensal orthologs of the human autoantigen Ro60 have recently been shown to activate human Ro60 autoantigen-specific CD4 memory T cell clones, further supporting the notion of human T cell cross-reactivity with commensal antigens (54).

To address experimentally the potential role of commensal bacteria in APS and SLE, Ruff et al. (53) treated (NZW × BXSB)F1 hybrid mice with broad-spectrum antibiotics (vancomycin or ampicillin), and showed that depleting gut microbiota decreased anti-β2GPI antibody levels and prevented thrombotic events in this model (53). SLE-related autoantibodies (anti-dsDNA and anti-RNA) and mortality were also diminished in antibiotic-treated (NZW × BXSB)F1 hybrid mice (55). From a pathophysiologic perspective, it was noted that (NZW × BXSB)F1 hybrid mice had impaired gut barrier function compared to non-autoimmune C57BL/6 mice. Loss of barrier function culminated in bacterial growth within the mesenteric veins, mesenteric lymph nodes, liver, and spleen. Full-length 16S ribosomal DNA sequencing of single colonies from organ cultures of (NZW × BXSB)F1 hybrid mice detected *Enterococcus gallinarum*, a Gram-positive gut commensal of animals and humans. Antibiotic treatment of the mice suppressed translocation of the microbiota, and correlated with reduced levels of T cells (Th17 and T follicular helper cells) and T cell cytokine signatures, as well as autoantibody levels and immunopathology. Of note, Manfredo Vieira et al. (55) also found *E. gallinarum* in liver biopsies from SLE patients, and showed that stimulation of primary non-autoimmune human or murine hepatocytes with *E. gallinarum* induced the production of β2GPI and type I interferon. These investigators (55) propose that translocating commensal bacteria may act in a number of ways to incite or perpetuate autoimmunity, including molecular mimicry, Th cell differentiation skewing, and induction of autoantigens (e.g., β2GPI) in colonized tissues. As Manfredo Vieira et al. (55) did not investigate whether β2GPI-reactive T cells were suppressed after microbiota depletion in their animal model, it is not clear whether antibiotic treatment impacts β2GPI-reactive T cells specifically. However, their findings showing increased β2GPI production in *E. gallinarum*-colonized liver and decreased anti-β2GPI autoantibody production in antibiotic-treated mice support a potential role for microbiota in the APS- and SLE-related manifestations observed in (NZW × BXSB)F1 hybrid mice.

### β2GPI-Reactive T Cells in Atherosclerosis

#### Non-APS-related Atherosclerosis

Relatively little is known about β2GPI-reactive T cells located within tissues, as compared to those found in peripheral blood. Profumo et al. (57) evaluated β2GPI-reactive T cells in patients with carotid atherosclerosis, both those occurring in peripheral blood and those infiltrating advanced carotid atherosclerotic plaques. The study population comprised 35 consecutive patients undergoing endarterectomy for symptomatic carotid artery stenosis or asymptomatic severe or pre-occlusive carotid-artery stenosis (≥70%). Individuals with recent infections, autoimmune diseases, malignancies, and inflammatory diseases prior to enrolment were excluded from the study. Only 1 patient was positive for anti-β2GPI and anti-CL (IgM for both antibodies). Plaque-derived and peripheral blood T cells were analyzed in 5 patients, while only peripheral blood T cells were analyzed in the remaining 30 patients. A proliferative response to native β2GPI was observed in 1 of 5 (20%) plaque-infiltrating T cell isolates, and in 8 of 35 (23%) PBMC samples, compared to no response in PBMCs from 13 healthy controls. β2GPI-reactive T cells in both plaque and PBMCs produced elevated IFN-γ and TNF-α, and were predominantly Th1-polarized. The importance of these findings lies in the occurrence of β2GPI-reactive T cells among atherosclerotic patients, despite the absence of overt autoimmune disease.

#### APS-Related Atherosclerosis

Benagiano et al. (58) also studied β2GPI-reactive T cells located within atherosclerotic lesions, but included patients with APS. CD4 T cell clones were generated from atherosclerotic lesions of 4 aPL-positive patients with primary APS and 4 aPL-negative individuals, all with arterial occlusive disease of the lower extremities. Thirty-two of 115 (28%) CD4 T cell clones from primary APS patients proliferated in response to native β2GPI, as compared to none of 263 CD4 T cell clones from aPL-negative individuals. CD8 T cell clones from the same lesions did not respond to β2GPI. All β2GPI-reactive plaque-derived T cell clones expressed IFN-γ and TNF-α in response to β2GPI, consistent with a Th1-like phenotype. More than 80% (26 of 32) of the β2GPI-reactive T cell clones recognized an epitope within Domain I, while only 19% (6 of 32) recognized an epitope within Domains IV and/or V. Interestingly, the predominance of Domain I-specific T cell clones in atherosclerotic lesions of primary APS patients differs from the predominant Domain V specificity observed in peripheral T cells of APS patients (34, 35, 38). Of note, the β2GPI-reactive T cell clones induced expression of tissue factor and matrix metalloproteinase-9 by autologous monocytes, and promoted total IgG, IgM, and IgA production in autologous B cells. In addition, plaque-derived β2GPI-reactive T cell clones were able to induce perforin-mediated cytotoxicity in EBV-transformed B cells and Fas/Fas ligand-mediated apoptosis in Jurkat cells, suggesting their ability to cause cellular damage.

A second group (42) also demonstrated an immunodominant T cell epitope within Domain I of β2GPI, in this case in PBMCs of 9 patients with APS (primary or secondary not specified). Five of the 9 patients had a thrombotic event, while the remaining 4 had fetal loss. The patients were all positive for IgG anti-CL and anti-β2GPI. In addition to recognizing recombinant Domain I/II, CD4 T cells responded to reduced, but not native, β2GPI. The authors identified an epitope located in Domain I of β2GPI (within the leader sequence of β2GPI [^1^MISPVLILFSSFLCHVAIAG^20^]), and showed that it is recognized in the context of MHC class II haplotype DRB3^*^02:02.

Benagiano et al. (58) have speculated on the mechanistic role of β2GPI-reactive T cells in atherothrombosis. They hypothesize that endothelial cells and professional antigen-presenting cells within the atherosclerotic plaque may become targets of the cytotoxic and apoptotic activity of β2GPI-reactive T cells, resulting in necrotic cores characteristic of unstable atherosclerotic lesions and leading eventually to atherothrombosis. Conti et al. (44) have shown that PBMC proliferation to β2GPI is associated with a history of arterial thrombosis and with increased intimal-medial thickness among patients with SLE and primary APS. They suggest that β2GPI-specific T cell reactivity may be associated with subclinical atherosclerosis. Of note, β2GPI has been found in human atherosclerotic plaques (59). Similarly, β2GPI was found in early murine atherosclerotic lesions, and co-localized with macrophages, endothelial cells, and smooth muscle cells in atherosclerosis-prone mice (60). Furthermore, immunization with β2GPI promoted enhanced fatty streak formation in atherosclerosis-prone mice (32, 61), and transfer of β2GPI-reactive T cells promoted early atherosclerosis (60). Finally, Domain I-specific antibodies have been shown to be more strongly associated with thrombosis and obstetric complications than antibodies to other domains of β2GPI (62, 63).

### β2GPI-Reactive T Cells in Murine Models of SLE

Our group has developed a murine model of SLE that is induced in healthy non-autoimmune mice by immunization with heterologous β2GPI and a strong pro-inflammatory stimulus (lipopolysaccharide [LPS]). Disease in this model bears striking similarities to human SLE (16). Notably, the specificities and sequential emergence of SLE-associated autoantibodies in this model closely mimic those seen in human SLE (7). The production of autoantibodies culminates in the development of overt glomerulonephritis (16). Furthermore, we have shown that β2GPI-reactive T cells are critical for the development of this model, and they are associated with the development of SLE autoantibodies across a spectrum of MHC class II backgrounds (64, 40). While epitope specificity of the β2GPI-specific T cell response is related to MHC class II haplotype, mice of multiple haplotypes develop SLE-related autoantibodies (40). A common T cell epitope was shared across different MHC class II haplotypes and therefore may be important in the development and spread of the autoimmune response (41). Specifically, peptide 31 (amino acid sequence ^165^LYRDTAVFECLPQHAMFG^182^) in Domain III of β2GPI was recognized by T cells in both C57BL/6 (I-A^b^) and C3H/HeN (I-A^k^/I-E^k^) mice immunized with β2GPI and LPS. This epitope was also recognized by T cells from MRL/MpJ-*Tnfrsf6*^*lpr*^ (MRL/lpr) mice, which develop murine SLE spontaneously. Despite recognizing a common epitope, β2GPI-reactive CD4 T cells from the induced and spontaneous models differ in cytokine production: T cells from the induced model expressed IFN-γ (Th1-like), while T cells from MRL/lpr mice expressed both IL-17 and low levels of IFN-γ (Th17-like) (41). Together, these data demonstrate the sharing of a β2GPI-reactive T cell response by both induced and spontaneous models of SLE, and raise the intriguing possibility that this T cell response mediates epitope spread of autoantibodies in both models.

β2GPI-reactive T cells from the induced SLE model also recognize a Domain II epitope (peptide 23, amino acid sequence ^111^NTGFYLNGADSAKCT^125^) that is shared by both murine and human T cells. Unlike peptide 31 in Domain III that is recognized across MHC class II haplotypes in mice, this peptide appears to be recognized only by MHC class II I-A^b^-bearing murine T cells (e.g., from C57BL/6 and 129S1). However, human CD4 T cell clones from a patient with primary APS (40) responded to this peptide in the context of a single HLA-DR allele, DRB1^*^04:03, an allele that has been associated with the presence of aPL (both anti-CL and anti-β2GPI) in a European cohort of SLE patients (65). These findings further point to a potentially similar β2GPI-specific T cell response in SLE and primary APS.

**Figure 1:**
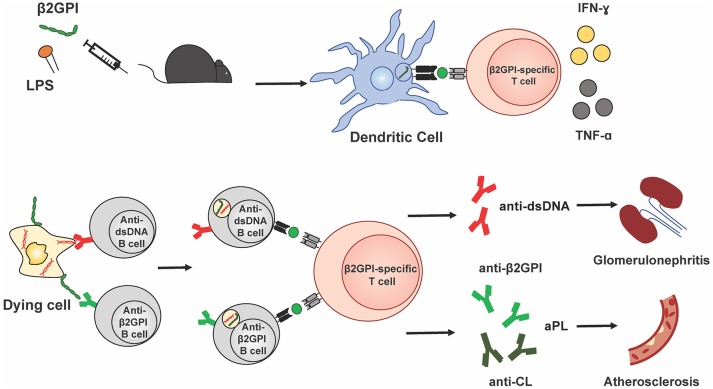
β2GPI-reactive T cells promote autoantibody production and pathology in APS and SLE. This simplified schematic diagram illustrates a possible mechanism by which β2GPI-reactive T cells may promote the development of multiple serological and clinical outcomes. Immunization of mice with β2GPI and lipopolysaccharide (LPS) results in the presentation of β2GPI-derived peptides (green circles) to T cells by dendritic cells and activation and proliferation of β2GPI-reactive CD4 T cells. In the second phase of the response, β2GPI-reactive CD4 T cells could provide help not only to B cells specific for β2GPI, but also to other autoantigen-specific B cells. We propose that autoantigen-specific B cells can recognize their cognate antigen on dying cells and ingest dying cells that have β2GPI bound to their surface. This would result in the presentation of β2GPI peptides in the context of MHC class II on the B cell surface, and allow T cell help from a β2GPI-reactive CD4 T cell. The examples shown here are a B cell specific for the SLE autoantigen dsDNA, and a B cell specific for β2GPI, with β2GPI-reactive T cell help resulting in anti-dsDNA and anti-β2GPI (and/or anti-CL), respectively. In addition, pro-inflammatory cytokines (e.g., IFN-γ and TNF-α) produced by the β2GPI-reactive T cells can impact other cells and tissues, either locally in a paracrine manner or at a distance in an endocrine manner. Depending on the autoantibodies and cytokines produced, different pathologies would arise (e.g., thrombosis or atherosclerosis with aPL, and glomerulonephritis with anti-dsDNA). In this way, β2GPI-reactive T cells could be a driving force for autoantibody production and pathology in both APS and SLE.

Taken together, these data suggest that β2GPI-specific T cell specificities in murine SLE, both spontaneous and induced, overlap with those found in human SLE and APS. They further indicate that, at least in mice, β2GPI-reactive T cells are associated with the development of multiple and diverse autoantibodies in SLE. Figure 1 illustrates a possible mechanism by which β2GPI-reactive T cells could promote the development of multiple serological and clinical outcomes. According to this scenario, β2GPI-reactive CD4 T cells provide help to autoantigen-specific B cells that have taken up apoptotic (or other dying) cells and present MHC class II-bound β2GPI peptides on their surface (16, 24). In this manner, B cells specific for various SLE-associated autoantigens (expressed on the surface of dying cells) can receive T cell help, and secrete class-switched autoantibodies against these autoantigens (including anti-β2GPI, anti-CL, and anti-dsDNA). In addition, pro-inflammatory cytokines produced by the β2GPI-reactive T cells can impact other cells and tissues, either locally in a paracrine manner or at a distance in an endocrine manner. Depending on the autoantibodies and cytokines produced, different pathologies could arise (e.g., thrombosis or atherothrombosis with aPL, and glomerulonephritis with anti-dsDNA). Through this mechanism, β2GPI-reactive T cells could be a driving force for autoantibody production and pathology in both APS and SLE.

## Concluding Remarks and Future Perspectives

The nature and role of β2GPI-reactive T cells in APS and SLE remains a field ripe for future investigation. The current literature provides evidence that β2GPI-reactive T cells are critical to the pathogenesis and pathophysiology of APS. While less solid, evidence also exists for a similar role of β2GPI-reactive T cells in SLE. The presence of class-switched IgG aPL in SLE-prone individuals almost a decade before disease onset suggests that β2GPI-reactive T cells are present in these individuals early in the disease process. The association of aPL with earlier disease onset, as well as a more complex and severe clinical course, further supports the potential importance of these T cells in SLE. β2GPI-reactive T cells are also found in both non-autoimmune and autoimmune patients with atherosclerosis, either subclinical (44) or overt (44, 57, 58). This last finding highlights the clinical relevance of β2GPI-reactive T cells in patients other than those with APS and SLE. Experimental findings in murine models of APS (64, 66, 67), atherosclerosis (32, 60), and SLE (40, 41) complement these human data and strengthen the notion that β2GPI-reactive T cells play an important role in the pathogenesis of these diseases.

Despite these advances, many key questions require further investigation. Further comparisons of β2GPI-reactive T cells from patients with primary vs. secondary APS, as well as from SLE patients with vs. without APS, are needed to determine whether differences in T cell specificity contribute to differences in clinical course between these patient groups. Moreover, careful analyses of the associations between β2GPI-reactive T cells and autoantibodies other than aPL, particularly in SLE patients, are required to establish the role of these T cells in B cell epitope spread. Given the difficulties inherent in human studies, murine models of SLE become critical. For example, determining whether a β2GPI-reactive T cell response is found in spontaneous models of SLE other than MRL/lpr mice would help to establish whether this is a common mechanism for the development of SLE-like autoimmunity. T cell epitopes found to be important in the murine models could then be evaluated in human SLE, and their mechanistic role elucidated by genetic manipulation of the various murine models.

The nature of the antigen recognized by β2GPI-reactive T cells is, of course, critical in any study of these T cells. To date, it has often been difficult to compare studies because of methodological differences in the nature of the antigens used (e.g., native vs. chemically modified) and the lack of epitope mapping in many studies. Despite the practical limitations, studies would include ideally both native and reduced β2GPI, as well as complete epitope mapping using recombinant fragments and peptides encompassing the entire sequence of β2GPI. As ethnicity and HLA restriction likely play an important role, HLA genotyping would be extremely helpful in these studies. Finally, careful comparison of β2GPI-reactive T cells in tissues (e.g., atherosclerotic plaques or nephritic tissue) vs. the peripheral blood of the same individuals would illuminate potential differences in the specificity and function of these T cells.

β2GPI-reactive T cells clearly play a role in APS and SLE, but an improved mechanistic understanding of their contribution to clinical outcomes is needed to render these cells useful diagnostically and/or as therapeutic targets. Equally important, elucidation of the epitope specificity of β2GPI-reactive T cells should provide insight into the nature of the initiating stimulus for these T cells. Finally, an appreciation of whether β2GPI-reactive T cells are involved in promoting epitope spread to non-aPL autoantibodies will further our understanding of how multiple autoantibodies arise in SLE.

## Author Contributions

Manuscript was drafted by JR and DS, and edited by DS, RS, MK, JL, and JR.

### Conflict of Interest Statement

The authors declare that the research was conducted in the absence of any commercial or financial relationships that could be construed as a potential conflict of interest.
